# Experiences of supporting older persons in completion of an exercise and nutrition intervention: an interview study with nursing home staff

**DOI:** 10.1186/s12877-021-02039-7

**Published:** 2021-02-05

**Authors:** S. Vikström, H. K. Grönstedt, T. Cederholm, E. Franzén, G. Faxén-Irving, Å. Seiger, A.-M. Boström

**Affiliations:** 1 Department of Neurobiology, Care Science and Society, Division of Occupational Therapy, Karolinska Institutet, Stockholm, Sweden; 2 Stockholms Sjukhem R&D unit, Stockholm, Sweden; 3 Allied Health Professionals, Function Area Occupational Therapy & Physiotherapy, Karolinska University Hospital, Stockholm, Sweden; 4 Department of Geriatric Medicine, Uppsala University Hospital, Uppsala, Sweden; 5 Karolinska University Hospital, Theme Aging, Stockholm, Sweden; 6 Department of Public Health and Caring Sciences, Clinical Nutrition and Metabolism, Uppsala University, Uppsala, Sweden; 7 Department of Neurobiology, Care Science and Society, Division of Physiotherapy, Karolinska Institutet, Stockholm, Sweden; 8 Department of Neurobiology, Care Science and Society, Division of Clinical Geriatrics, Karolinska Institutet, Stockholm, Sweden; 9 Department of Neurobiology, Care Science and Society, Division of Nursing, Karolinska Institutet, Stockholm, Sweden

**Keywords:** Nursing home, Staff, Adherence, Health intervention, Sit-to-stand, Oral nutritional supplementation, Interview, Experience, Expectation, Well-being

## Abstract

**Background:**

The interactions between nursing home (NH) staff and their residents are crucial not only for the atmosphere at the NH but also for achieving care goals. In order to test the potential effects of daily physical activities (sit-to-stand (STS) exercises) combined with oral nutritional supplementation (ONS), a randomized intervention trial (the Older Person’s Exercise and Nutrition (OPEN) Study) was performed in NH residents. One aspect of the study was to interview and report the NH staff’s experiences of supporting the residents in fulfilling the intervention.

**Methods:**

In this qualitative study, individual and focus group interviews were performed in eight NH facilities with NH staff who had assisted residents in performing the 12-week ONS/STS intervention. An interview guide developed for this study was used to assess staff experiences of the intervention and its feasibility. The transcribed interviews were analyzed inductively following a constant comparative method and with input from experts in the area, described in Grounded Theory as a reliable technique for researchers to form theory and hypothesis in unexplored areas.

**Results:**

Three main themes relating to the health-promoting intervention emerged. These included: 1) insights into attitudes towards health in general and NH care specifically; 2) intervention-related challenges, frustrations and needs, and 3) aspects of collaboration and opportunities.

The overarching hypothesis derived from the analysis reads: A health-promoting intervention such as the OPEN-concept has great potential for integration into NH life if a combined empathic and encouraging attitude, and a structure to keep it sustainable, are in place.

**Conclusions:**

NH staff experienced the health-promoting intervention as a potentially positive concept, although it was suggested that it works best if introduced as a general routine in the unit and is integrated into the daily planning of care.

**Trial registration:**

ClinicalTrials.govIdentifier: NCT02702037. Date of trial registration February 26, 2016. The trial was registered prospectively

**Supplementary Information:**

The online version contains supplementary material available at 10.1186/s12877-021-02039-7.

## Background

Nursing home (NH) residency is commonly associated with a passive lifestyle as most of the residents spend their daily lives in a seated or lying position. This passive lifestyle results in lower physical capacity regarding balance, walking speed and aerobic endurance [1]. In addition, physiological complications, such as constipation, pressure ulcers and reduced muscle mass, are reported [2]. As the age at NH admission increases, residents face a higher risk of frailty, dependency and an increased risk of falls [3]. A NH study in Sweden indicated that, for example, balance can be improved by training, but also that reduced physical exercise can lead to a rapid decline in fitness in less than 3 months [4]. Admission to a NH is strongly related to decrease in mobility, which in turn affects quality of life [2, 5].

Geriatric research has given increased attention to health promotion in older people [6, 7] and has identified that health-promoting self-care initiatives are dependent on sustained physical function [8]. This is a prerequisite for an active and social daily life [6]. Studies show that increased physical function of residents in NHs can be reached if the exercise is adapted to the ability and aspirations of each individual resident. It was reported in 2016, for example, that frail older residents benefit most from short, repeated training sessions [9], while a NH study found correlations between social and physical activities and a sense of thriving among residents [10]. A common finding in several of the studies referenced is, that frail older persons are in need of a high level, individually tailored support, which requires knowledge, time and attention from the staff.

The Older Person’s Exercise and Nutrition study (OPEN) comprised a 12-week intervention for residents in NHs consisting of sit-to-stand exercises (STS) four times daily and the intake of a protein-rich oral nutritional supplement (ONS) twice daily [11]. The research staff introduced the study and engaged the staff working in the NH units where the participating residents lived in considering plans for how to give individual support during the intervention. Lunch seminars were held where the staff were asked to reflect on the individual residents and on the situations and places in the NH where the STS exercises could be performed. They were encouraged to use a person-centered approach and discuss with each older person, in order to identify how to do the exercises and when to serve the ONS during the day. The results from the OPEN study indicated that participants who had high adherence to the combined interventions increased their chair rise ability compared to the control group [12]. Further, interview findings from *NH residents* in the OPEN study revealed their enthusiasm to collaborate with staff and that the intervention was an easily performed and adaptable concept that empowered residents and added meaning to their day at the NH [13]. Thus, a potential for health promotion initiatives is identified.

However, sub-optimal conditions for the implementation of new knowledge and changes in working routines have recently been identified in NHs, while the increased number of residents with severe care needs challenge NH economies [14]. It is primarily persons with extensive needs, such as severe cognitive and/or physical impairments, who are considered eligible for admission to NHs [15]. The increased workload has also resulted in decreased possibilities for attracting permanent staff to NHs [15]. With high staff turnover, the possibilities of adopting new knowledge are challenging, especially with the unpredictable and often urgent nature of NH care [14, 16]. In a large quality improvement study, staff identified several improvement areas where they with collaboration and sharing of experiences could improve the life of residents at their NH unit, despite time-constraints being an obstacle [17]. Staff also illustrated genuine compassion for the residents and showed to take it upon themselves to safeguard the NH residents against interventions they perceived as potentially negative for some [18]. These findings indicate, improvements need to be person-centered and staff needs to be convinced of their benefits.

Strategies to improve the quality of care in NHs commonly include development of national guidelines for care [7, 19]. Such guidelines exist for persons with dementia in Sweden [7], where both physical activity and person-centered care is strongly recommended [20]. An increasing number of studies show that NH residents have the possibility to maintain or even boost their strength by integrating physical activities with an adequate nutritional intake [12, 21]. However, since the integration of health-promoting NH interventions needs to consider both work-related challenges and person-centered care it is of interest to explore staff experiences of the OPEN study indepth. Our research question thus read: How would a health-promoting concept, based on collaboration between NH residents and staff work in daily care from a staff perspective? Thus, the aim of this qualitative study was to describe the experiences of NH staff concerning supporting the residents in their completion of the combined intervention.

## Methods

### Study design

This qualitative interview study with a constructivist approach and exploratory design was part of the OPEN study [11], an RCT trial performed in 62 units in eight NHs. Of the 102 participants allocated to either an intervention group (IG) or a control group (CG), the 52 IG group participants (mean age 85.8 years, mean MMSE score = 18 out of 30) performed the 12-week combined intervention supported by staff. The staff were asked to help adapt the OPEN components to the needs and aspirations of each resident. No significant improvement in physical function was measured, although 21 high adherence persons increased their fat-free mass and either maintained or improved their physical function [12].

Post-intervention, the ordinary day and evening shift staff at units who cared for at least one IG participant were invited to individual interviews (*n* = 10) or focus groups (*n* = 15 in 4 groups). Three participants in the individual interviews and less than half of the participants in the focus groups spoke Swedish completely fluently. Of the 25 participants (21 women, 4 men), 22 were nurse assistants and 3 were nurses.

In this interview follow-up study we chose a Grounded Theory (GT) approach as described by Charmaz [22], since this constructivist approach, where researchers also learn about the research foci during data collection, fits the explorative design of the study. The interview questions focused on the participants’ experiences of supporting the NH residents who had chosen to perform the 12-week health-promoting intervention.

The GT approach by Charmaz [22] is reinforced by the epistemology of constructivism and interpretative theoretical stance. In this, the researcher is viewed as being part of the study rather than totally excluded. The constructivist approach appreciates multiple realities and their complexities, rendering visible experiences within the situations and relationships that are hard to see at first glance. Thus, the focus of the theory developed within the constructivist approach also depends on the interpretations of the researchers.

### Research team and reflexivity

The interprofessional OPEN research group consists of an occupational therapist (SV, first author), a nurse (AMB, OPEN PI), two physicians (TC, ÅS), two physiotherapists (EF, HG) and one dietitian (GFI) (gender distribution F = 5 & M = 2). All authors have a PhD and held combined clinical and academic positions within the field of geriatrics at the time of the study. All were part of the 3-year planning and formulation of the study. They actively applied for grants and were updated on the literature in the area. The interviewers (SV & HG) introduced the OPEN concept to the participants (with GFI). The main author (SV) did not have any other established relationship with the staff who knew her only as an independent researcher related to the OPEN intervention. The interviewer is an occupational therapist who does not include physical exercise, such as STS, or nutritional supplements as part of her professional repertoire and could thus be considered reasonably non-biased.

### Setting

Eight NH units from two communities in Sweden were represented in the study. They were all small-scale units where each resident had a combined living room and bedroom with a cooking corner, and a bathroom. The shared facilities typically included a sitting room(s), kitchen and dining area, and the management and rehabilitation quarters were located adjacent to the units. All NHs used the concept of designated main carer (DMC) whereby a staff member has the task of being extra attentive to a specific resident to gain in-depth knowledge and provide person-centered care. The eight NHs were enrolled in the study over a period of 1.5 years and the time-period for the interviews was from May 2016 to December 2017.

### Sampling and recruitment

The OPEN concept was introduced at 2–3 lunch seminars per NH unit to staff from all levels of the organization, i.e. nurse assistants (NAs), registered nurses (RNs), rehabilitation staff and managers. Staff members were also encouraged to reflect on how to support the residents. Despite explicit requests from management to participate, only about 2/3 of the day and evening staff did so. A few weeks after the interventions and follow-up testing of the NH residents, purposive sampling [23] was used to recruit staff from eight NHs with experience from the intervention for interviews in a secluded room at the NH in question. Night-shift staff, temporary staff, rehabilitation professionals and managers were therefore excluded. Ten NAs with the role of DMC for at least one participating resident were interviewed individually (by SV & HG). Four focus group interviews with NAs and RNs with knowledge of the health-promoting intervention were also performed (by SV).

### Data collection

In total, 10 individual interviews and 4 focus group were performed with NH staff who had experience of assisting residents in performing the 12-week ONS/STS intervention. An interview guide was used to assess staff experiences of the intervention and its feasibility (See additional File 1). Inductive analysis of the transcribed data (by SV) followed an iterative constant comparative method, while also receiving input from members of the project group who are experts in the area; this is described in GT as a reliable technique for researchers to form theory and hypothesis in unexplored areas.

The GT approach allows for data collection to be iterative, letting the early gathering of data and analysis inform and shape continued data collection [24]. Although a semi-structured interview guide concerning the participants’ experiences of supporting NH residents in the OPEN concept was used, information retrieved in early interviews was subsequently incorporated in follow-up questions. For example, one staff member described the ONS as ´treats´ given after performing STS. This resulted in the additional question: How were the ONS presented to the residents?

GT also opens up for diverse data-sets, which was helpful because low staffing levels, in combination with a challenging NH unit layout, made it hard to recruit staff to individual interviews. Focus group interviews held at staff meeting times were therefore a complement in our endeavor to obtain rich and nuanced data. However, the interview guide was still of use, although with fewer follow-up questions, since the interviewees built on each other’s answers. For example, different voices described the support strategies they had used in unit D which made us ask for descriptions of how they had given support. Both sets of interviews were recorded and averaged 45 min in length. These were then transcribed verbatim as closely as possible, together with the field notes [22]. Data collection ceased when saturation, i.e. repetitions of previously gathered data, occurred [24].

### Data analysis

Due to the iterative nature of the GT approach [22] and the lengthy data collection period, we initiated the analysis after the first set of interviews. The transcribed interviews were coded line-by-line, where aspects of staff experiences of supporting older residents in the combined intervention were marked keeping as close as possible to the participants own words. During the analyses, codes were constantly compared, aiming to identify unique but diverse groups, i.e., emerging subthemes.

The closeness to each respondent’s phrasing helped us to compare similarities and differences within and across the data. Allowing the richness of data – with a vast number of citations- to be maintained throughout the analysis contributes to the results section and buffers against the preconceptions of the researcher skewing the data [22].

According to Charmaz [24], coding occurs in stages. In our initial coding, we viewed the data inductively and generated as many insights as possible from early data, keeping codes close to the original data. In a parallel focused coding, we pursued a selected set of central codes that were most prevalent or important in the analysis. These were *encouraging attitudes* and *health-integration initiatives* in the daily care. We then re-examined the data to find all available insights regarding these codes. Three main themes in the results were distinguished under which we, after performing further constant comparatives, described sub-themes. Second opinions on the data from the highly competent research team with their diverse experience-based knowledge were important in this step to confirm that the data were shaped by the respondents [23].

Our tentative theoretical hypothesis is expressed as a set of concepts that are related to each other in a cohesive way and account for the data that were collected [24]. From a trustworthiness perspective it is worth noting that the developing hypothesis has been presented to specialist audiences (e.g. EUGMS Berlin), as well as to participant groups, and was found to be accepted and to resonate with their experiences, indicating transferability [22].

## Results

Three main themes relating to the combined intervention emerged (See Table 1). These included 1) insights into attitudes towards health in general and NH care specifically; 2) intervention-related challenges, frustrations and needs; and 3) aspects of collaboration and opportunities. Quotes from staff are labelled A-H to indicate the unit they worked in. Abbreviations are used to indicate quotes from individual interviews (Ii.) and fieldnotes (Fn). Where no such indication is given, the findings derive from a focus group.

**Table 1:** Themes and subthemes

**Attitudes towards the health-promoting intervention and its feasibility in the NH setting** *Different understandings of the health-promoting intervention* *Empathic and person-centered attitudes* *Need to take responsibility and create enthusiasm*	
**Intervention-related challenges, frustrations and needs** *Fear of lacking time or putting pressure on residents* *Having realistic views about the needs that could be met* *Sense of responsibility to do well that could create tension* *Need for a model that motivates staff long-term*	
**Aspects of collaboration and opportunities** *Develop care using new support strategies* *Integrating the ONS/STS concept works best* *Leaders must create conditions for continuity*	

### Attitudes towards the health-promoting intervention and its feasibility in the NH setting

Analyses showed how staff had ***different understandings of the health-promoting intervention*** and that not all staff members had taken it to heart. Several reactions at project initiation were positive: *“It’s great to address muscles and the mobility of older persons. The exercises are simple and we can encourage them, because they* *do* *sit still a lot!” (*D). *“I am convinced that one’s mental capacities can be positively influenced by exercise” (*A). Although the DMCs were specifically targeted, a few displayed surprisingly limited insights: *“I missed the info-meeting and felt overwhelmed. It was mentioned as important, but I never grasped why” (*D Ii.).

The diverse range of understanding could result in low ambitions to support residents, as in this example where a different chair would have benefitted the older person: *“Her own chair is very low and hard to get up from, so she perceives the training as a strain” (*D, Ii.*).* Skewed perceptions of the intervention concept - where repeated daily sessions of STS exercises were requested - also prevailed: “*She sits and sleeps much of the day. But when she wakes up, we might try to help her move her arms and legs a bit.”* (B). Some lacked conviction regarding the need for the protein-rich ONS, claiming the food served was already enriched. *“All residents gain weight when they move in here”* (A). *S*ometimes the ONS was exchanged for something else, which meant compromising the study. One DMC equated the ONS with similar drinks: *“For example yoghurt smoothies. They are more fatty, but we try to serve all residents the same to avoid any mix-ups”* (E). A few units diverted from the twice daily provision and gave the ONS as a late evening, after-dinner drink or as a substitute for a bedtime sandwich. “*We serve them late, and in the morning, so as to not spoil their appetite”* (C).

Views concerning the results of the intervention for the residents also spanned from being potentially mentally invigorating; *“She seems slightly more alert now”* (E), to increasing physical function such as walking or general strength: “*They* *do* *seem to grow stronger!*”(A).

The analysis also bore examples of ***empathic and person-centered attitudes***: *“People move here in their last days of life. So interventions need to be adapted to suit each person*” (C).

The ONS distribution was often individually adapted, with consideration given to, for example, how it was served, flavor and whether it was chilled or not. Most participants appreciated the glasses with measuring marks that motivated the residents to drink up and provided an overview of intake: “*She is a picky eater, so we monitor her intake”* (D). Some staff referred to the ONS as a tasty reward after the STS exercises, and others as a health-promoting ONS.

It was apparent that empathy and efforts to provide person-centered care varied. One RN underscored the creativity of NAs: “*They solve tricky situations and adapt things for each resident*”. A NA replied *“I try to facilitate many STS, for example standing to climb out of their night gown; pulling up underwear, trousers; stepping into their shoes, and then sitting down in-between”* (A). Person-centeredness was practiced less with more independent residents: “*Well, I guess he did the STS. He did some in the dining room at least”* (C). “*He signed up but didn’t do it for long*. *He’s a bit...lazy, if I may say so”* (F). Similarly, two groups of staff had less empathic attitudes: “*Not all of us are interested in enthusing residents to move. It’s not part of our job description”* (A). *“Not all staff are equipped to make the extra effort as this is challenging”* (D)*.*

Most participants, especially the DMCs, felt the ***need to take responsibility and create enthusiasm*** to embed the intervention into the daily care: *“We talked a lot about it. We feel confident in caring but are much less used to supporting exercises”* (A, Ii). Some NAs described it as an extra burden: *“We need to stand beside them…and count” (*B). *“Harder than it sounds”* (A). *“It needs some extra effort”* (C). The support that was required varied too: *“My lady can’t cope on her own. We have to help”* (B). “*He’s reminded when we show up on his doorstep. He trained extensively, if reminded...” (B).* One group took responsibility by way of progress monitoring: *“She gained two kilos. Her clothes fitted her like before, so she must have built muscles”* (D). Other groups were less structured: “*Our staff did it differently. It depended on who worked that day as it was up to each staff member to pursue the OPEN concept”*(C).

If participants declined a suggestion to do STS, staff described yielding to their wish, despite being aware of the person’s previous interest in the intervention: *“We adapt to the motivation of our residents”* (C), *“The older man I am DMC for was offered the possibility to do the STS, but often declined. I consider one daily STS to still count”* (A). Such attitudes to health promotion, where reluctant residents lacked support, occurred in most NH units. Other units showed how they had integrated the combined intervention into their daily work: *“We were all on board”* (C). “*I have convinced my colleagues to continuously remind him to do the STS” (*D Ii.). *“Counting is as important as enthusiasm and we encouraged them a lot. But when there’s no longer a need, our previous care pattern might return. It’s a shame …”*(C). In contrast, one unit described plans to further the combined intervention: *“We’ll create even more customized care plans, where each resident’s motivations are addressed” (*D).

### Intervention-related challenges, frustrations and needs

The analyses further identified reoccurring reluctance from staff that related to a ***fear of lacking time or putting pressure on residents***. When managing the intervention, the staff had to both plan the coaching to fit into the day and then perform it with the residents. Some wanted a more flexible concept: *“Mornings are always very busy as residents constantly interrupt you” (*F). This was especially the case in small-scale NHs. “*One of our residents trained independently and reported to us, which was positive. Others needed constant support”* (page 12)(D, Ii.). *“We genuinely want it to work, it is so close to our hearts, no matter the abilities of a resident. We work 8-hour days, with nine residents. There* *should be* *time”* (G, Ii.).

Staff were also reluctant to put pressure on residents to fully complete the combined intervention period. A ‘yes’ at the start of the project was not interpreted strictly; *“Taking the ONS and doing STS [for 12 weeks] is what’s requested, but you can’t force them. At least we haven’t”* (A). Others prioritized being generous: *“Our unit resembles a family where the residents are precious. If she asks me for an ONS, I reply that she’s had it. -But they are so delicious! Then she gets one. Fine by me”* (D, Ii.). Great satisfaction was also expressed when there was no need for pressure and the STS exercises were performed spontaneously: *“Then it’s nice to be part of spreading joy in the unit*” (A). Sometimes it was the residents who encouraged the staff to do the combined intervention: *“It was a bit hard for us to follow. But* *he* *found it rewarding and shared his achievements, which gave us inspiration. Super!*” (C).

Staff also expressed the importance of ***having realistic views about the needs that could be met***. For example, the intervention opened up for the creation of either individual or group solutions: “*The only chance for group STS is at the meal table”* (F). *“One option for STS is when they are anyway all gathered”* (C). *“It creates a nice atmosphere and is good for you. They struggle together and have fun!*” (A). Others viewed dining room exercises as interrupting the calm atmosphere: “*It can stir up emotions and possibly jeopardize nutritious food intake*” (Fn F). *“In dementia units, things seldom go according to plan”* (C).

A common obstacle that was mentioned concerned perceived joint- and/or muscle pain. “S*he didn’t do well as her pain increased” (*F). “*He did them for a while, but his hip hurt too much*”(A). Staff described trying to overcome such obstacles. Some found it an advantage to give the ONS with medications prior to the STS exercises. Residents could also benefit from mental preparation: *“We approach them prior to the actual visit to plant a seed: - I’ll be back soon to support you with your exercise!”* (A) *“Our strategy is to catch them after breakfast: -OK ladies, it’s time to do STS! We join them long enough to keep their spirits high”* (H).

Findings revealed particular challenges for DMCs whose ***sense of responsibility to do well could create tension.*** Frustrations occurred in their attempts to reach high adherence to the combined intervention: “*As we work irregularly, colleagues had to substitute for us. But project charts weren’t signed so I had to push colleagues to do it*” (A). *“My colleagues were reluctant. I had to ask: Did she do the STS? Drink the ONS?”* (G, Ii.). “*In one way, it’s nice that it’s over, as I was responsible and colleagues didn’t comply” (A).*

One DMC had given up: “*I hope the regimen was followed. Signatures probably got lost among the rest of the tasks”* (C). One unit reduced internal tensions by encouraging residents to take charge: “*As he improved, we asked him to walk and request his ONS from the kitchen”* (F). However, field notes from that same unit revealed that less mobile residents missed out on several ONS servings. Some yielded to the challenge of others’ different views to avoid tension: *“Individual staff members reason differently, also concerning their perceptions of residents’ needs”* (D, Ii.). One group reflected on the possible benefits of having one staff member with extra knowledge and motivation to push staff to take equal responsibility for residents´ health needs.

In addition, staff described a **need for a model that motivates them long-term**. A RN shared her conclusions: *“The mind-set needs to be planted in each staff member right from the start. And everyone needs to raise his or her level of ambition. Sadly, it is easy to divert from what we know is good, although long-term integration of it might bring positive social side effects too” (*A). Another unit reflected similarly: “*Changing mind-sets is no quick-fix. The inclusion of exercises needs to be learned in a playful way”* (C). Another voice: “*Yes. And hide it in the daily routines”* (C). A third voice: *“But still tell them that STS is good exercise!”* (C).

One group suggested a future alternative for embedding a similar combined intervention in NH routines: *“Maybe all residents could receive a small ONS shot. They should* *all* *have that possibility. If all residents become more alert, that could raise the ambitions of the staff too, to support STS*” (A). Four focus groups described the combined intervention as a positive experience that they embraced: *“Because the STS is anyway easy to incorporate during dressing, or at meal times. Just move the chair out a bit and do some*. *Maybe it should be a national care model? So that no matter which NH one works in as a NA or RN, this concept would be viewed as important - because it is!”* (C).

### Aspects of collaboration and opportunities

Data showed that, to some extent, the OPEN concept had helped staff to ***develop care using new support strategies***. Previously, residents with fewer care needs could be neglected: *“The project gave us an excuse to regularly visit a man we had avoided bothering before. We found him to be very isolated! Our visits to coach him in STS gave benefits from both respects”* (D). A common denominator in units where the OPEN intervention was perceived as successful was staff/resident collaboration to fine-tune the support that was needed. This was then communicated broadly and made a unit-wide routine. “*It was time-consuming, but we also got to see the benefits of prioritizing exercise and protein”* (B). *“Now we are all used to it and it gets done, even if I am not there to assist”* (D Ii.).

When successfully integrated, the ONS was also discussed among staff who shared the same views: *“She takes the ONS in one serving and describes it as a good size that is filling”* (D)*.* A colleague added: *“Yes. She likes them. They are just right”* (D). The quotes also illustrate a new stance among the staff of not merely performing practical care, but also taking on a role as coach. “*We’re spreading joy. A nice role for staff, encouraging the residents”* (A). Some also adopted a staff-resident interplay that worked: *“She says: I have done my training! I reply that I’m proud of her. That gives her the energy to continue” (*G). One unit summarized their development thus far: “*It won’t happen at once, and it can vary from one day to another, but we’ve started to develop skills for seizing opportunities. And we noticed that the residents slowly got used to us asking them to exercise”* (G).

One insight that was shared was that ***integrating the ONS/STS concept works best***. This involves building on old knowledge and integrating new. For example, some reported that their former experiences with ONS had to be re-evaluated. *“Although we’ve used ONS before, then it was for persons who were malnourished, or persons with dysphagia. So ONS are not new, but the target group and the rationale is”* (D, Ii.). Some described strategies for integrating the combined intervention: “*I’ve made a box with a folder for each resident so that nothing gets lost” (*A). Another new approach used was for NAs to collaborate closely with RNs. “*Our tiny lady had such trouble taking the ONS. But the RN and I prolonged the time available by starting early in the morning with repeated encouragements to drink*” (B).

The integration of the OPEN concept affected staff differently. *One* person responded: *“I would say we get positive energy from this. Seeing smiles on the residents´ faces is contagious. But full integration is hard”* (F). Some staff described reasons for not integrating the concepts relating to their own lack of communication skills. They claimed to lack the vocabulary and correct pronunciation to be able to provide effective support, especially to residents with dementia. “*They don’t understand me when I ask them to sit and stand, so that’s one reason why I didn’t succeed”* (G). The primary success factor staff mentioned was integration of the intervention into a strict daily routine. *“Working in a similar way, all of us. Taking on a health-promoting approach that is incorporated in the broad scheme of things on our unit”* (C).

The analysis showed that unit ***leaders must create conditions for continuity***. One unit, where the staff perceived themselves as being successful, praised their RN: “*She gave us clear instructions right from the start and made sure we understood. She even sat down with us individually. Then it was done without any friction” (*D Ii.). Another unit referred to the leaders’ well-functioning structure: *“At our daily planning meeting we identified needs and inserted them into the activity folder. Such structure gives us good continuity”* (A). One DMC shared her insights: “*Staff have positives and negatives. We use our differences, but it takes some effort, and everyone needs to do something extra*” (G Ii.). Other groups planned to discuss the future with their managers: *“We have warmed to this now and will discuss if we should continue”* (D). *“We have concluded that the concept is beneficial for the staff too. Exercise is good for you” *(B). *“We’ve done it ourselves! A colleague came to our meeting and told us to do the STS for two minutes as a micro-break”* (H).

### Hypothesis building from the analyses

A GT approach with its iterative ways of capturing phenomena is a trustworthy tool to form credible hypotheses in unexplored areas. From these findings, a tentative hypothesis would be that *a health-promoting intervention such as the OPEN concept has great potential to be integrated into NH life if a combined empathic and encouraging attitude, and a structure to keep it sustainable, are in place.*

## Discussion

With reference to the above hypothesis, we identified that **staff motivation is a crucial factor in the intervention’s success** when integrating a health-promoting intervention such as the OPEN concept. One of the key prerequisites for this is having a combined encouraging and empathic attitude, i.e. providing **person-centered support to enable activities**. In addition, the working structure might need to move towards adding **health-promoting care** to the nursing care provided in order to keep a health-promoting intervention sustainable. Hence, the findings are discussed from these insights: 1) Staff motivation as a crucial factor in the intervention’s success, 2) Person-centered support to enable activities, and 3) Health-promoting care and work-related challenges.

### Staff motivation – a crucial factor in the intervention’s success

One key finding was that staff motivation was a crucial factor for most residents to achieve success when pursuing the concept, especially for persons with severely reduced mobility or dementia. Staff motivation seemed important for planning, initiating and pursuing the support. In previous studies, supervision and structured outcome monitoring have been identified as critical components for the sustainability of interventions amongst staff [25–27]. In the OPEN intervention, the achievement scores of each resident for performing STS and drinking the ONS (supported by staff) were collected by the head nurse on a weekly basis. As revealed in Vikström et al. [13], lack of access to their score progress was experienced as demotivating by the participating residents. This could possibly have similar effects on the staff who provided the daily care. Furthermore, findings by Colon-Emeric [28] underscore that individuals are more likely to follow regimens when the potential outcomes are clearly stated. In our study, we aimed for the OPEN components to be adapted to the needs and aspirations of each resident. Consequently, there was no clearly stated way or “one-model-fits-all” recommendation. This too might have reduced motivation among the staff.

Where successful adjustments to the combined intervention were made to fit the resident’s individual preferences, there seemed to be a shared agreement between the staff and the resident. Some residents also seemed to have been the driving force in the activity or had stated the individual goals they wanted to reach with support from the staff. These findings concur with interview findings from the older residents in the OPEN study, suggesting that residents achieving a goal through provision of ONS and exercises - tolerated by the older residents and easily administered - may also act as a staff motivator [13]. Hence, the older residents’ wishes needs to be clearly stated and continuously repeated to all staff involved.

### Person-centered support to enable activities

The Swedish National Guidelines for Care of Persons with Dementia suggest collecting life stories on admission to NHs in order to increase person-centered care. These include memories and experiences, but also aspirations for future activities at the NH [7]. However, the performance plan that staff members set up for their residents rarely includes segments where future aspirations, such as creative activities or exercise, are captured. This might explain the staff’s reactions to the health promoting ambitions of the OPEN concept, for example that they described being reluctant to prioritize supporting and reporting due to time limits or a feeling that the requests for training were unrealistic. Attitudes might have been more aligned with the OPEN requests if the residents’ life story aspirations had been part of the care plan.

Based on the findings in this study, there might also be reason to believe that the fundamental idea of the OPEN concept – requesting staff to support health promotion among frail, but motivated residents - stands in contrast to, or even in disagreement with, contemporary attitudes among staff within Swedish nursing homes. Staff hesitated or even omitted to coach residents who declined exercise despite residents having earlier expressed an interest. The reason given was to avoid putting pressure on the residents, but this might stem from the staff not feeling confident enough to coach residents or feeling that they lacked specific guidance, since we aimed for solutions that were person-centered.

Since the launch of the Swedish National Guidelines for Care of Persons with Dementia in 2010, the promotion of physical activity in older persons with dementia has been highlighted as extremely important [7]. However, as shown above, supporting a person’s remaining capability can be difficult if a combination of staff being less proficient in the country’s native language and residents having extensive care needs prevails. In our study, most of the residents had cognitive impairments or dementia. A recent study suggests that an effective way to reach a sustainable change in the staff’s approach is to provide them with regular coaching in how to give specialized dementia care [27]. Our findings also concur with a recent study describing the struggles that care staff perceive when asked to transform from being caregivers to becoming enablers so that residents can manage everyday activities [29]. Encouraging coaching to increase independence might therefore be necessary to fulfill the goals of health promotion in NHs.

### Health-promoting care and work-related challenges

The OPEN concept was ambitious in that it requested diverse groups of staff to understand, embrace and integrate the concept, while also convince and encourage the residents. The findings resonate with an ethnographic NH study on culturally mixed groups of staff showing that language barriers led to less effective communication between staff and less progressive working environments [30]. Similar findings by Schilgen et al. [31] showed that intercultural staff groups had divergent understandings of the aims and goals of NH care, which impeded collaboration within the work force [31]. The high proportion of temporary staff is a potential limitation of the study and might explain why large numbers of staff seemed to have missed the information meetings. This might also explain the limited engagement of some respondents. The findings concluded that staff considered the concept to be unrealistic for some colleagues to undertake, claiming that they lacked the ability to coach and engage residents.

Our findings illustrate that units whose management adopted a ‘staff-centered’ approach seemed to have been most successful, for example the group with the RN who held one-to-one meetings with each staff member to ensure they understood the intent. Parallels are found in a similar Canadian study also illustrating staff benefits of hands-on support [21]. Such a staff-centered approach from management might also be fruitful in ensuring quality improvement, which is welcome in this era of constant shortages of educated care staff in NHs [32, 33].

In this study, the regular staff were introduced to the concept through seminars and were reminded by their managers. We were inspired by the idea of *blended facilitation* which Pimentel et al. [34] has shown to be useful for quality improvement in NHs. Our findings also resonate with a study showing that sustained interventions requiring coordination among staff members are highly influenced by factors within the organizational context, external support and the feasibility of the intervention itself [35]. In our view, if adjusted to the unit’s organization and supported by the management, the easy-to-adopt OPEN concept has the potential to reach prominent health-promoting gains. Although interventions for frail older persons are difficult to control for [36], it has been argued that provision of support to manage everyday life might be beneficial for both the persons with dementia and for society [37]. Golinowska et al. [6] and the National Board of Health and Welfare in Sweden have suggested that interventions to strengthen physical function among older persons is a fundamental health-promoting element in the achievement of cost-effective care [7].

### Methodological considerations

This qualitative study provides valuable insights into how a health-promoting intervention in NHs might be perceived by the staff. In the qualitative analysis, the data from the 25 staff members proved to be rich enough to reach data saturation, with new interview responses and unique perspectives ceasing to appear [24]. To secure the trustworthiness of the findings, fellow researchers in the research group reflected on the findings throughout the period of analysis [23]. These experienced researchers in the field of geriatrics have all been able to recognize and mirror the data, thus confirming its dependability [23]. It should be noted that qualitative research allows people’s stories to be told, although only as fragments of thoughts. This is especially pertinent when respondents, such as some of the respondents in our study, perceive restrictions in giving responses when communicating in a language that is not their mother tongue. In addition, some respondents had received information about the OPEN study “second hand” through the interpretation of a colleague rather than at the information meetings [23].

## Conclusions

The combined intervention of high protein oral nutritional supplementation and exercise is experienced by nursing staff as a positive concept. Our findings suggest that staff engagement in NH interventions benefit from viewing each older resident in a person-centered way. This includes a changed perspective among staff to identify intrinsic abilities and potential driving forces within each older resident, which contrasts with commonly prevailing views that NH residents have low willpower and are at the end-stage of their lives. Our study indicates that a concept that is broadly integrated as a routine in the unit and aims for increased physical function, integrated into the daily planning of care, where managers are staff-centered, could be a strong building block for reaching elements of health-promotion in daily NH care.

## Supplementary Information

**Additional file 1.**

## Data Availability

The datasets generated and/or analyzed during the current study are not publicly available as individual privacy could be compromised but are available from the corresponding author on reasonable request. For data on OPEN trial see study protocol in BMC Geriatrics [11] and the OPEN trial publication [12].

## Abbreviations

OPEN: Older Person’s Exercise and Nutrition
STS: Sit-to-stand
ONS: Oral nutritional supplementation
DMC: Designated Main Carer
NA: Nurse assistant
RN: Registered nurse
