# Supplementary material for: Experiences of supporting older persons in completion of an exercise and nutrition intervention: an interview study with nursing home staff
Source: BMC Geriatr. 2021 Feb 5;21:109. doi: 10.1186/s12877-021-02039-7 (PMC7866449; doi:10.1186/s12877-021-02039-7)
Supplement: Supplementary file 1 — Additional file 1. [file 12877_2021_2039_MOESM1_ESM.docx]

# **Interview questions posed to staff in the OPEN study**

## **Introduction**

### This set of questions were created solely for the OPEN study evaluation and have (to our knowledge) not been used elsewhere.

### As participation was volontary and no personal details about the participating staff was obtained, they thus stayed anonymous to the interviewing researcher apart from their first names (never used in transcripts), the *Regional Ethical Review Board in Stockholm*, D no. 2013/1659-31/2, 2015/1994-32 and 2016/1223-32 approved interviews with recorded verbal consent only.

## **Activities performed at the start of each interview session**

### Repetition of the information that this interview will be part of the research performed in the OPEN project, with its focus on NH residents performing sit-to-stand in combination with intake of protein-rich oral nutritional drinks.

### The researcher underscore that participation is volontary, and that the data will be handled confidential, and that they themselves and quotes used will be anonymous.

### Each participant is then asked to give a verbal consent to their participation, which is recorded on tape prior to the start of the interview.

## **Questions**

### 1) Ask how the staff was informed about the project and the initial thoughts on supporting NH residents to participate in the combined intervention to do sit-to-stand and take oral supplements.

### 2) Ask what the staff think the perceptions and experiences of the NH residents are on participating in the project.

### 3) Ask staff to describe what they themselves think and feel about supporting and encouraging *sit-to-stand* exercises (for individual interview: as a contact person)?

### 4) Ask them about details/examples from the floor. How did it work to integrate *sit-to-stand* in the daily life at the ward? Describe how the NH residents did the exercises and what affected the outcome?

### 5) What have they notice about the elderly's perception of performing the *sit-to-stand* (incl. amount, support, location etc.) and how and when in the day they are taken, and how come (if relevant)?

### 6) Ask them to describe what they themselves think and feel about supporting and encouraging *nutritional supplements* (for individual interview: as a contact person)?

### 7) Ask them about details from the floor. How does it work to integrate *nutritional supplements* in the daily life at the ward? Describe how the resident took the nutritional supplement and what affected the outcome?

### 8) What have they notice about the elderly's perception of drinking them (incl. taste, texture, amount, administration etc.) and how and when in the day they are taken, and how come (if relevant)?

### 6) Ask for reflections on what they perceive or experience you can gain from participating in the study, and what findings they have noticed (if any)?

### 7) Ask for thoughts on how to perform similar interventions in the future. Recommendations they might have for training or administer drinks in a different way, including individual vrs. group, or in different settings or routines?
